# RBD, sexsomnia, sleepwalking, and sleep paralysis comorbidities: relevance to pulmonary, dental, and behavioral sleep medicine

**DOI:** 10.5935/1984-0063.20210018

**Published:** 2021

**Authors:** Carlos H. Schenck

**Affiliations:** Minnesota Regional Sleep Disorders Center, Departments of Psychiatry, Hennepin County Medical Center and University of Minnesota Medical School, Minneapolis, USA

## INTRODUCTION

This issue of *Sleep Science* contains three reports^1^^-^^3^ on diverse REM and NREM parasomnias that serve as illuminating entry points to a broad range of comorbidities that are interlinked with the parasomnias, with relevance to multiple sleep medicine subspecialties. Besides the fascinating mechanistic questions raised by this interlinking, there are also important clinical management issues that need to be considered. Parasomnias, to a surprising extent, are situated at the core of sleep medicine. The International Classification of Sleep Disorders, 3^rd^ Edition (ICSD-3)^4^ recognizes that instinctual behaviors can be pathologically released with the parasomnias, involving locomotion (sleepwalking), aggression (RBD and NREM parasomnias), eating (sleep related eating disorder), and sex (sexsomnia) that carry the potential for adverse physical, psychological, and interpersonal consequences.

**Figure 1:**
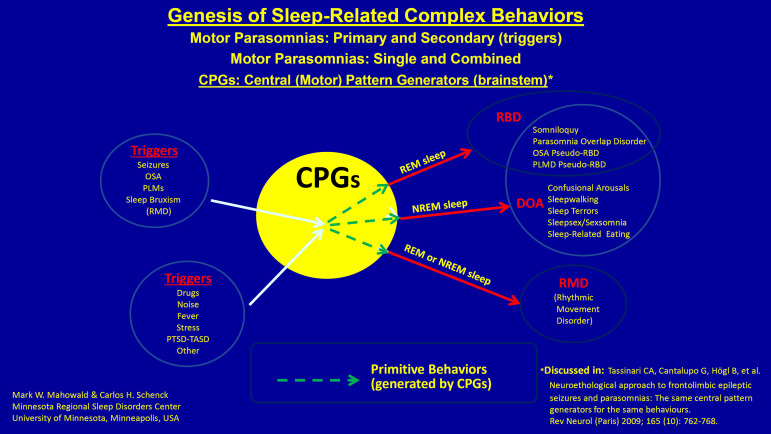
Framework for understanding parasomnia behavioral release and their triggers.

Central pattern generators (CPGs) in the brainstem, subserving primitive behaviors, are inappropriately activated with the parasomnias. Figure 1 provides a useful and scientifically sound framework for understanding parasomnia behavioral release and their triggers, based on the seminal work of Tassinari et al. (2005)^5^ and Tassinari et al. (2009)^6^ from Bologna related to CPGs, parasomnias, and nocturnal seizures. In regards to the NREM parasomnias, the factors that predispose, prime and precipitate these parasomnias have been comprehensively reviewed by Pressman (2007)^7^.

## RBD AND OSA

The report in this issue by Giardino et al. (2021)^1^ provides further data to support the compelling term “respiratory REM sleep without atonia (RWA) benefit” on comorbid idiopathic RBD (iRBD)-OSA. In this novel retrospective case-control study of 25 RBD-OSA patients and 26 patients with REM-predominant (or exclusive) OSA without RBD, the authors found that the RBD-OSA group had a significantly lower drop of the SpO_2_ nadir of nearly 7%, indicating that the RWA with RBD had lessened the severity of the OSA. The protective effects of RWA against OSA in iRBD were also found in another case-control study by Jo et al. (2019)^8^ that included both REM and NREM sleep OSA for the analyses. During REM sleep, AHI and RDI measures were significantly lower in RBD-OSA patients compared to OSA patients. Overall, the protective effects were more potent in the supine position compared to the non-supine position. Also, the prevalence of REM-related OSA was lower in RBD-OSA patients than in OSA patients (9% vs. 33%). Another study of patients with RBD, OSA, and PD found that RBD lessened the severity of OSA^9^. However, patients with combined RBD-OSA-PD had the most severe cognitive impairment, revealing complexities of this clinical scenario.

Six years after our group formally identified RBD, in 1986, we published an abstract with descriptive data that raised the question of whether RBD protected against OSA^10^ (which Giardino et al. (2021)^1^ mentioned). We cited a study on an experimental cat model of RWA-RBD produced by dorsolateral pontine lesions that found enhanced diaphragmatic ventilation during REM sleep^11^. Although respiratory timing parameters were not altered by the lesions, the inspiratory rate of rise was significantly increased in all cats, and the brief pauses (40-100ms) in the diaphragmatic EMG normally seen in REM sleep were virtually abolished. These experimental findings could provide a basis (besides others) for the findings in the study of Giardino et al. (2021)^1^.

The literature on RBD-OSA is rapidly expanding. A good starting point for interested readers would be the just-published critical review on all the known OSA-RBD associations by Jung and Oh (2021)^12^ from Seoul entitled: “*Is REM sleep behavior disorder a friend or foe of obstructive sleep apnea? Clinical and etiological implications for neurodegeneration.*” A few years previously, the Wing group from Hong Kong critically addressed the same issues^13^. They commented on how RBD is highly comorbid with OSA, as Gabryelska et al. (2018)^14^ have also identified - with the added suggestion that CPAP therapy of OSA might also improve self-reported RBD symptoms, in addition to standard RBD treatment. This observation merits systematic study. Additional important questions include: i) Is there a threshold level of RWA% of total REM sleep needed to initiate benefit for OSA in REM sleep?; ii) Does the extent of RWA in RBD correlate with the extent of improvement in OSA measures during REM sleep, and is it a linear relationship?; iii) What are the interactions in respiratory and muscular activities with OSA and RWA in RBD?; iv) Does increased REM motor phasic activity in RBD, besides RWA, also play a role in improving OSA measures during REM sleep?

Another OSA-RBD relationship involves “OSA pseudo-RBD” that was formally identified by the Barcelona group, involving older men with severe OSA (mean AHI=67) who, besides snoring and EDS, had violent dream-enacting behaviors that were documented by video-PSG (vPSG) to emerge with apneas, and with REM-atonia being preserved, thus confirming the absence of RBD^15^. Successful treatment of OSA with CPAP also controlled the dream-enacting behaviors (DEB), as confirmed by spouse history and by repeat vPSG, with preserved REM-atonia reconfirmed.

The same Barcelona group also identified severe “PLMD pseudo-RBD” that also involved older men with violent dream-enacting behaviors. video-PSG (vPSG) documented a median PLMI=61 with aggressive behaviors triggered by the PLMs in NREM and REM sleep, and with REM-atonia being preserved^16^. Therapy with pramipexole controlled the PLMD and the associated DEB, as confirmed by spouse history and by repeat vPSG, with preserved REM-atonia reconfirmed.

### Sexsomnia: 8 cases documented by vPSG in the literature

The documentation of sexual behaviors during sleep with vPSG serves several useful purposes, including convincing the patient (who usually is amnestic about this sleep behavior), and also validating the observations of the spouse/other bedpartner. Also, the reporting of these documented cases in the peer-reviewed literature highlights the legitimacy of sexsomnia as a *bona fide* medical sleep disorder, and secures its position among the NREM parasomnias, viz. confusional arousals and sleepwalking, as contained in the ICSD-3^4^. It also can facilitate the referral to behavioral sleep medicine specialists for interventions focused on the negative psychosocial consequences of the sexsomnia, as discussed in the report by Toscanini et al. (2021)^2^ in this issue.

Six of the 8 vPSG documented cases of sexsomnia reported in the literature involved males, with an age distribution of 16-49 years, including a male who had one episode of sexual intercourse, initiated by his wife’s touch during light NREM sleep^17^, and five males who had 35 documented episodes of sleep masturbation (range: 1-15 episodes per patient)^18^^-^^21^. There was no associated dreaming during these vPSG documented sexual behaviors during sleep. Triggers for the sexsomnia episodes included spontaneous NREM sleep partial arousals (n=3), OSA (n=1), sleep bruxism (n=1), and combined OSA-sleep bruxism (n=1). One adolescent patient had 4 episodes of sleep masturbation triggered by OSA in the first vPSG study, and then after the OSA was fully treated with CPAP, he had 4 more episodes of sleep masturbation triggered by NREM partial arousals in a second vPSG study^20^, exemplifying the complexity of sexsomnia in some clinical cases. This case finds a striking parallel to a complex parasomnia case of combined NREM parasomnia and severe OSA (AHI=39) involving a 55 y.o. man with a 20 year history of self-biting during sleep requiring surgical interventions, whose parasomnia control was only achieved with combined bedtime clonazepam therapy and BIPAP therapy^22^.

The first vPSG documented female with sexsomnia was a case of parasomnia overlap disorder^4^^,^^23^, with a total of 5 motor parasomnias in NREM and REM sleep^24^. This 60 y.o. married woman had presented for sleep evaluation on account of a 4 year history of RBD (confirmed by vPSG with increased EMG tone during REM sleep), with violent behavior towards her husband during dream-enactment. She also had a childhood-onset, lifelong sleepwalking and sleep talking history, along with episodes of sleep-related eating; vPSG documented an episode of sleep masturbation lasting 2-3 minutes arising from N3 sleep, with subsequent amnesia and no dream recall.

The second documented female with sexsomnia was 42 y.o married woman with 4 episodes of masturbation emerging from N2 and N3 sleep that was reported in this issue of *Sleep Science*^2^. Besides the critical vPSG documentation of sexual behaviors during sleep, this case illustrates the major adverse marital and familial consequences from the sexsomnia, including her masturbating during sleep in the marital bed while uttering the name of another man, and also including her young son hearing her moaning out loud sexually during sleep. There was no evidence suggesting an extramarital affair, illustrating how people have no control over their behavior or vocalizations during sleep (which is also recognized legally). Of note is that this is the second reported case of a married woman with sexsomnia who uttered the name of another man while masturbating in sleep with her husband laying next to her, but also without any evidence of an extramarital affair^25^. Toscanini et al. (2021)^2^ discuss the range of psychosocial problems associated with sexsomnia (as reflected in the title), and urge a multi-disciplinary approach to patient management, which can include consultation with a behavioral sleep specialist.

### Sexsomnia, RBD, OSA, sleep bruxism, and oromandibular myoclonus

Two cases of sexsomnia associated with OSA have been reported in which sustained control of both conditions was achieved with mandibular advancement device (MAD) therapy of the OSA^26^^,^^27^. There had been previous reports of CPAP therapy of OSA also controlling the comorbid sexsomnia, as reviewed^28^^,^^29^. Therefore, it is the control of the OSA, regardless of the therapy, that is crucial for the control of the secondary sexsomnia resulting from apnea-induced confusional arousals. The first case utilizing MAD therapy involved a 27 y.o. married man who presented for possible sleep apnea, and during the evaluation he also reported a 3 year history of sexual behaviors during sleep in which he attempted to disrobe his wife and initiate sexual intercourse, for which he had no recall^26^. There was a remote history of sleepwalking during childhood. PSG documented OSA with an AHI=6.9 and REM AHI=16.1. The patient refused CPAP, and MAD therapy was initiated at 50% maximal protrusion. Repeat PSG documented treatment efficacy, with an AHI=1.2. At one-year follow-up, full control of sexsomnia had been maintained, along with all OSA symptoms. The second case involved a 37 y.o. married man who presented with loud snoring^27^. His wife disclosed a history of sexual relations during sleep on a nightly basis for many years without the patient’s awareness. PSG revealed an AHI=15 with O_2_ saturation nadir of 91%. MAD therapy completely controlled the sexsomnia (and OSA symptoms) for 5 months, and then he switched to CPAP therapy because of jaw pain. Control of sexsomnia (and OSA symptoms) was fully maintained with CPAP at 6-month follow-up. Also, there were major interpersonal issues with his wife related to the sexsomnia, as discussed in the report.

The first two cases of vPSG-documented sexsomnia (masturbation) associated with sleep bruxism (SB) were reported from a multidisciplinary group at Wrocław Medical University, Poland^19^. Both cases involved men who had presented with multiple sleep complaints - but not including sexsomnia. The first case involved a 49 y.o. man with longstanding severe sleep and wake bruxism. Given his history of snoring, SB, poor sleep quality, frequent nightmares, and daytime fatigue, he underwent vPSG that documented 8 episodes of sleep masturbation lasting from several seconds up to 20 seconds during N1, N2, and REM sleep, but not from N3. There was no sexual climax, and no subsequent recall of the sexual activity. Of note was that each sexual episode was preceded by SB lasting at least a few seconds, with cortical EEG arousal. Clinical OSA was not documented, with an AHI<5. The patient had never been aware of any sexsomnia. He refused therapy of his SB.

The second case involved a 39 y.o. male with complaints of loud snoring, nightmares, muscle cramps during sleep, daytime fatigue, and erosion of his teeth; vPSG documented complex interactions among overlapping OSA, SB, and sexsomnia, with 15 prolonged episodes that followed a typical repeating sequence of video and PSG recorded abnormalities. One sequence lasted 17 minutes that was subdivided into repeating episodes that consisted of an O_2_ drop to 88% triggering a spontaneous arousal followed by SB onset, and after 3sec masturbation was initiated with the dominant left hand, lasting 20sec before abrupt cessation with the onset of an apnea episode. The 15 episodes of sleep masturbation occurred during N1/N2 sleep and almost always were triggered by SB, and the masturbation stopped when the O_2_ saturation reached a level of 89-90%. There was never climax and never any recall of the sexual activity. All episodes occurred from the supine position. Most SB events were associated with respiratory events and spontaneous arousals. The AHI was 33.5/hr, with average oxygen desaturation drops of 6.1%. CPAP therapy showed a good first night response, and additional treatments were proposed, but follow-up information was not provided.

SB has also been interlinked with RBD, along with oromandibular myoclonus (OMM), indicating the existence of another set of RBD/parasomnia comorbidities. In one study, PSG data were collected from 28 vPSG-confirmed RBD patients and 9 age-matched controls^30^. Patients were divided into two groups: 13 patients with iRBD and 15 patients with PD-RBD. Rhythmic masticatory muscle activity, a marker of SB, and OMM were scored. The rhythmic masticatory muscle activity index was found to be significantly higher during REM sleep in iRBD subjects compared to controls. A sleep laboratory diagnosis of SB was made in 25% of all patients. Patients with iRBD had significantly more OMM during REM sleep than controls. The authors concluded that in the presence of a high frequency of rhythmic masticatory muscle activity during REM sleep, RBD may be suspected, with further assessment being warranted.

Finally, SB can be part of a subclinical RBD symptom complex^31^. A 59 y.o. man underwent vPSG for the complaint of SB, and 5 episodes of SB (lasting 40-60 sec) were documented - all occurring during REM sleep, along with movements of his head, hands and fingers during REM sleep, accompanied by vocalizations. Excessive phasic EMG activity in multiple muscle sites were documented in REM sleep, together with bursts of REMs There was no clinical history of RBD.

### Sleepwalking, OSA, and prolonged sleep paralysis

The third parasomnia report in this issue of *Sleep Science* involved a 42 y.o. man with the late onset of complex and injurious sleepwalking with subsequent amnesia, followed by the onset of prolonged partial SP arising from a dream and affecting both legs for up to 20 minutes^3^. There was no history suggestive of narcolepsy, although the REM-latency during vPSG was reduced at 40.5 minutes, which the authors attributed to chronic sleep deprivation. However, an alternative explanation would be that this shortened REM-latency was another marker of dissociated REM sleep, along with the prolonged partial SP. An added complexity to the case was OSA, with an AHI=22.4. The authors discuss and cite the relevant literature on how OSA can promote SW on account of recurrent abnormal apnea-induced arousals and sleep disruption. However, on a case-by-case basis in complex scenarios, it can be impossible to predict the driving force for SW and the optimal therapy, which this case illustrates: the patient could not tolerate CPAP therapy, and so his OSA was left untreated. Fortunately, he responded to low-dose bedtime clonazepam, with benefit maintained at 6-month follow-up in the full control of SW and SP. Presumably the mechanism of therapeutic action was a NREM sleep-stabilization effect induced by clonazepam, which has been demonstrated in iRBD patients^32^.

An analogous complex clinical scenario involving sexsomnia with POD and OSA resulted in therapeutic responses that could not be predicted beforehand, involving a 42-year-old male with good response to CPAP therapy of OSA - without any benefit for the sexsomnia, which did respond substantially to bedtime clonazepam therapy^33^. Finally, the authors of the case of sleepwalking, SP and OSA^3^ found no evidence supporting comorbid seizure disorder presenting as sleepwalking and/or SP (including the use of the FLEP scale), since a case has been reported of focal epileptic seizures mimicking SP (and satisfying ICSD-2 diagnostic criteria for SP)^34^. Furthermore, the case of a 10 y.o girl was recently reported in which a second opinion was sought for an epilepsy diagnosis that could account for multiple nightly episodes of partial awakenings while appearing confused, with speaking of non-sensical words before resuming sleep^35^. Prior antiepileptic therapies had been ineffective. The vPSG recorded two typical events of confusional arousals (NREM parasomnia) triggered by apneas; her AHI=9. Tonsillectomy and adenoidectomy was the definitive treatment, as at 3-month follow-up no further episodes were reported and snoring had stopped. Also, 48 hour EEG monitoring and brain MRI had been normal. Therefore, this case demonstrated the complex interplay of OSA, NREM parasomnia, and presumed diagnosis - and therapy - of nocturnal seizures in a child, with vPSG identifying the correct diagnosis.

In conclusion, the authors of the three parasomnia papers in this issue of *Sleep Medicine* should be commended for their astute clinical observations in the two case reports^2^^,^^3^ and for the novel research design in documenting the benefit for OSA during REM sleep provided by RWA in iRBD^1^, which have stimulated a broader discussion of parasomnia comorbidities.
